# Chronic Diseases

**Published:** 1875-12

**Authors:** E. H. Gibbs


### CHRONIC DISEASES.

BY E. H. GIBBS, M. D.

Chronic Diseases, are those that have passed the active or inflammatory stage. Strictly speaking, a disease is not curable, until it has passed this stage and become to a certain extent "chronic." The word means "time," and any disease that has had time to pass the active stage, "chronic." The tendency of chronic affections, is to recover; and nine out of ten will recover by proper attention to hygienic laws, without medicine. It would be imprudent, in case of a severe attack of illness, to trust recovery to nature, without availing ourselves of medical advice; without in fact ascertaining the nature and the extent of the trouble and the proper remedy. In most cases, the province of the physician should be, to give us this information, and to spare us from drugs. But when there exists inflammation of a dangerous character, active and prompt measures must be resorted to.

In such cases, delays are always dangerous. From neglect nearly all cases of "lingering death" arise. It is strange they should be so numerous, since in their incipient stages, they are always curable. Most chronic diseases are curable, when no change of structure has taken place which seriously affects a vital organ. If the heart has become enlarged and the valves do not perform their work perfectly, complete recovery is impossible; because there is a change which no art can remedy. But with care the patient may live comfortably to old age. Consumption in its first stage is curable; and even later when large cavities have formed in the lungs, permanent cures have been affected. Rheumatic and nervous affections are almost always curable at any stage. That so many cases remain uncured, is the fault of the sufferers.

To preserve our bodies, so far as possible in sound health is a duty; but there is no duty that is so much neglected. Disease is frequently permitted to make its inroads on the system, unchecked for months or years before competent medical advice is sought. The most charitable explanation of such neglect is that it arises from ignorance. There is no subject about which people seem so unwilling to inform themselves as their health. It is lightly prized until it is lost. A lecture upon health and the means of preserving it, would put an intelligent audience in a profounder sleep than a long, dull, after-dinner sermon, on a hot Sunday. They would only think about it seriously when the pangs of an *invited* disease came to torture them as with the instruments of the "Inquisition."

---

A HINT.—Whenever you stop your horse, especially in cold, windy weather, be sure to turn his tail to the wind, then the centre of the circulation will be in a measure protected from the immediate action of the cold Besides, the buggy or sleigh helps materially to break it off. This is a simple suggestion, yet, of more importance than at first might appear, and may prevent an attack of rheumatism or other disease.

